# Diagnostic Performance of Infrared Nasal Thermography for the Detection of Enzootic Nasal Adenocarcinoma in Goats

**DOI:** 10.3390/vetsci13040389

**Published:** 2026-04-17

**Authors:** Pablo Quilez, Marta Ruiz de Arcaute, Marcelo de las Heras, Delia Lacasta, David Guallar, Javier Balado, José María González, Carlos Hedman, Alfredo Benito, Héctor Ruiz, Aurora Ortín

**Affiliations:** 1 Animal Pathology Department, Veterinary Faculty, Instituto Agroalimentario de Aragón-IA2, University of Zaragoza, Miguel Servet 177, 50013 Zaragoza, Spain; pquilez@unizar.es (P.Q.); martarda@unizar.es (M.R.d.A.); lasheras@unizar.es (M.d.l.H.); dguallar@unizar.es (D.G.); jmgsovino@unizar.es (J.M.G.); hedman@unizar.es (C.H.); hectorruiz353@gmail.com (H.R.); aortin@unizar.es (A.O.); 2 Ruminant Clinical Service, Veterinary Faculty, University of Zaragoza, Miguel Servet 177, 50013 Zaragoza, Spain; 3 Quesos de Catí, Cooperativa, 12513 Catí, Spain; 4 Gabinete Técnico Veterinario, de las Cortes Catalanas 932, 08014 Barcelona, Spain; 5 Exopol S.L, Poligono Río Gállego D-14, 50840 Zaragoza, Spain; abenito@exopol.com

**Keywords:** enzootic nasal adenocarcinoma, goats, infrared thermography, diagnostic imaging, screening tool

## Abstract

Enzootic nasal adenocarcinoma is a contagious neoplasm of goats for which early antemortem diagnosis remains challenging under field conditions. This study evaluated the diagnostic accuracy of infrared nasal thermography for detecting the disease using histopathology as the reference standard. Eighty-six goats from a dairy herd with confirmed enzootic nasal adenocarcinoma were examined by infrared thermography one day prior to slaughter under standardized environmental conditions. Thermal images of the ethmoidal region were qualitatively assessed for asymmetry or focal hyperthermia. Following slaughter, all heads underwent systematic necropsy and bilateral histopathological examination. Twenty-three goats (26.7%) were histologically confirmed as positive with confirmation by RT-PCR (Reverse Transcriptase Polymerase Chain Reaction) from tissue samples. Infrared thermography showed a sensitivity of 82.6% and a specificity of 90.5%, with an overall diagnostic accuracy of 88.4%. Positive and negative predictive values were 76.0% and 93.4%, respectively. Agreement between thermography and histopathology was substantial (Cohen’s κ = 0.711; *p* < 0.001). Although thermography did not achieve the specificity of macroscopic post-mortem examination, its non-invasive and rapid nature supports its potential as a preliminary complementary antemortem screening approach, although its applicability at herd level requires validation in broader and more representative populations.

## Simple Summary

Enzootic nasal adenocarcinoma is a contagious cancer that affects the noses of adult goats. It causes trouble breathing, weight loss, and eventually leads to death. The disease is hard to spot early because the first signs are mild, and confirming it usually requires testing after the goat has died. This makes it difficult to control the disease within a herd. In this study, we tested whether infrared thermography, a specialized camera that measures heat, could help identify sick goats before they develop serious symptoms or die. We checked 86 goats from a farm with a history of this disease using the heat camera on their noses before they were sent to slaughter. Afterwards, we used lab tests and close-up examinations to see if the goats had the tumour. The heat camera correctly identified sickest goats and rarely mistook healthy goats for sick ones. The camera results matched the lab findings well. While this camera cannot fully replace lab testing, it offers a quick, gentle, and practical way to spot possible cases earlier, helping farmers and veterinarians better manage their herds.

## 1. Introduction

Enzootic nasal adenocarcinoma (ENA) is a malignant epithelial neoplasm of contagious nature that primarily affects the nasal glands of adult sheep and goats [1]. The disease is associated with a betaretrovirus known as enzootic nasal tumour virus (ENTV), for which two variants have been identified: ENTV-1, associated with sheep [2], and ENTV-2, responsible for cases in goats [3]. Experimental transmission has been confirmed in both species [4,5]. In goats, ENTV-2 has been detected in multiple tissues, including tumours, lymph nodes, kidneys, lungs, peripheral blood mononuclear cells and bone marrow [5], supporting systemic viral dissemination.

ENA is a chronic, progressive disease of the upper respiratory tract [6,7] reported worldwide, except in United Kingdom, New Zealand and Australia, although reliable epidemiological data remain limited. Prevalence within affected herds ranges from 0.1% to 15% [1,8]. Despite its global distribution and economic relevance, early diagnosis remains challenging, particularly under field conditions.

Clinically, ENA in goats follows a slow and progressive course. Early signs are often non-specific and include persistent serous nasal discharge, inspiratory dyspnoea, mouth breathing and progressive weight loss [1,8,9]. In advanced stages, craniofacial deformities may develop due to progressive destruction and remodelling of the frontal and maxillary bones, sometimes accompanied by exophthalmos. A characteristic feature is unilateral serous nasal discharge resulting in the typical “washed nose” appearance caused by chronic hair loss. By the time these clinical signs become evident, lesions are usually well established. Affected animals usually die as a result of secondary bacterial complications, including pneumonia or septicaemia [8].

Necropsy combined with histopathological examination of the ethmoidal region remains the gold standard for ENA diagnosis [10]. Histologically, ENA is characterised as a low-grade adenocarcinoma arising from the luminal epithelium of the nasal glands. Immunohistochemical staining for cytokeratins CK17 and CK18 has been used to support tumour cell identification [11]. However, these diagnostic approaches are inherently post-mortem and therefore unsuitable for early detection or herd-level control strategies based on antemortem screening.

A further limitation in ENA diagnosis is the apparent absence of a detectable humoral immune response. Naturally infected goats do not develop measurable antibodies against ENTV capsid or envelope proteins [1,12], precluding reliable serological testing and reinforcing the need for alternative antemortem diagnostic approaches.

Molecular assays have been developed as antemortem alternatives. Several PCR-based techniques, including RT-PCR and RT-qPCR targeting different genomic regions of ENTV-2, have demonstrated high analytical sensitivity and specificity in nasal swabs and tumour tissues. Apostolidi et al. [10] described a real-time RT-qPCR assay targeting the env/U3 region, whereas He et al. [13]. developed a SYBR Green-based RT-qPCR targeting the gag gene. An EvaGreen-based RT-qPCR assay has also been reported as a rapid and cost-effective method for clinical samples [14]. Nevertheless, these techniques require specialised equipment and trained personnel, limiting their practical application under routine farm conditions.

Imaging modalities have gained increasing interest as complementary diagnostic tools. Radiography, computed tomography, magnetic resonance imaging and thermography have been used to detect and characterise nasal lesions in small ruminants [15,16]. Among these, infrared thermography offers practical advantages: it is rapid, non-invasive, does not require anaesthesia and enables real-time assessment of surface temperature distribution. Previous studies have suggested that ENA-associated lesions may induce localised increases in surface temperature over the ethmoidal region [15,17]. However, thermographic findings may also be influenced by inflammatory or obstructive nasal conditions, highlighting the need for careful interpretation and validation against reference standards.

Although thermography has shown potential as a complementary imaging technique, its diagnostic accuracy for ENA detection in goats under field conditions has not been fully characterised. In particular, sensitivity, specificity and agreement with histopathological findings remain insufficiently defined.

Therefore, the aim of this study was to evaluate the diagnostic accuracy of infrared nasal thermography for the antemortem detection of enzootic nasal adenocarcinoma in goats, using histopathology as the reference standard. By estimating sensitivity, specificity and inter-method agreement, we sought to assess whether thermography could be incorporated into practical herd-level screening strategies.

## 2. Materials and Methods

### 2.1. Study Design and Ethical Considerations

An observational diagnostic accuracy study was conducted to assess the performance of infrared thermography for the detection of enzootic nasal adenocarcinoma in goats, using histopathological examination as the reference standard.

All animals included in the study were culled for productive or sanitary reasons unrelated to the objectives of this research. No experimental procedures were performed, and thermographic imaging was carried out during routine clinical handling prior to slaughter. Therefore, in accordance with national regulations, specific ethical approval was not required.

### 2.2. Animals and Study Population

The study was conducted in Murciano-Granadina goats from an intensive dairy farm located in the province of Castellón, Spain. The herd comprised approximately 800 animals managed under intensive production conditions and fed concentrate ration and straw ad libitum. In the years preceding the study, a sustained increase in ENA cases had been observed, with an estimated herd-level prevalence of approximately 7% based on clinical and post-mortem findings.

A total of 86 goats were included according to the availability of intact post-mortem head specimens and the absence of severe mechanical damage to the nasal structures during slaughter. Animals were enrolled consecutively among goats selected for culling, which may have introduced spectrum bias and potentially affected diagnostic performance estimates.

Of the 86 animals, 82 were females and 4 were males. Age at culling ranged from 0.5 to 9.1 years (mean: 3.6 years). Reasons for culling, as determined by the attending veterinarian, included mastitis (40.7%, *n* = 35), clinical suspicion of ENA (32.6%, *n* = 28), cachexia (8.1%, *n* = 7), reproductive disorders (5.8%, *n* = 5), lameness (4.7%, *n* = 4), advanced age (2.3%, *n* = 2), udder conformation defects (2.3%, *n* = 2), small ruminant lentivirus infection (1.2%, *n* = 1), accidents (1.2%, *n* = 1), and respiratory disorders (1.2%, *n* = 1).

### 2.3. Infrared Thermography

Infrared thermographic examinations were performed using a FLIR E5 Pro infrared camera (FLIR Systems, Wilsonville, OR, USA), which features a thermal sensitivity (Noise Equivalent Temperature Difference, NETD) of <0.06 °C (<60 mK) at 30 °C, enabling the detection of small differences in surface temperature. Thermal images were acquired one day prior to slaughter, either during milking or while animals were at rest, in order to minimise stress and movement artefacts during image acquisition.

Environmental conditions were standardised during the thermographic assessment. Ambient temperature ranged from 18 to 22 °C, and both direct airflow and solar radiation were avoided. Images were obtained from a fixed distance of 0.5 m using frontal and lateral views, with primary analysis based on the frontal view to ensure consistent visualisation of the nasal region. Camera emissivity was set to the manufacturer’s recommended value.

A standardised qualitative region of interest (ROI) was defined bilaterally over the external nasal area anatomically corresponding to the ethmoidal turbinates, the most common site of ENA development. The ROI was located in the dorsocaudal region of the nasal plane, extending cranially to the base of the nasal bone and caudally to the upper third of the nostrils, delimited medially by the nasal midline and laterally by the external nasal margin. Thermographic assessment focused exclusively on these ROIs to evaluate bilateral thermal symmetry and identify focal or diffuse areas of increased surface temperature.

**Figure 1:**
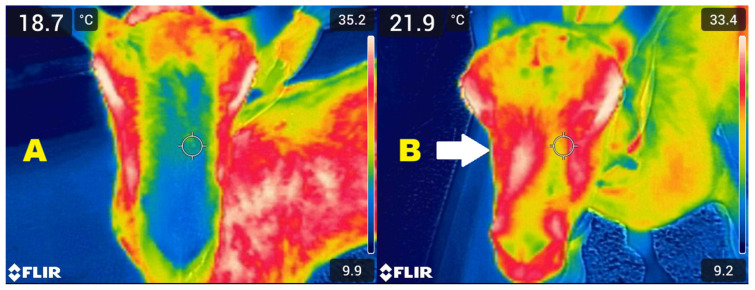
Infrared thermographic images of the nasal region in goats obtained in frontal view. (**A**) Clinically healthy animal showing symmetrical temperature distribution over the ethmoidal region. The cooler (blue) areas visible within the nostrils reflect physiological evaporative cooling associated with normal airflow. (**B**) Goat affected by ENA, exhibiting marked unilateral thermal asymmetry and focal hyperthermia over the ethmoidal region (arrow), consistent with underlying neoplastic involvement.

Thermal images were analysed using FLIR Ignite software (FLIR Systems; https://ignite.flir.com/) to generate thermal maps and evaluate temperature distribution patterns within the predefined ROIs. Image interpretation was qualitative and based on visual comparison between both nasal sides. Thermographic findings were classified according to three predefined criteria based on the identification of abnormal thermal patterns relative to expected physiological distribution, rather than strictly relying on contralateral comparison. A unilateral positive result was defined by the presence of focal hyperthermia or distinct thermal asymmetry within the Region of Interest (ROI) relative to the contralateral side (Figure 1), while a bilateral positive classification was assigned when focal hyperthermia was detected on both sides, regardless of symmetry. Conversely, a negative result was characterised by the absence of focal hyperthermia and the maintenance of a symmetrical thermal distribution.

Thermographic image acquisition was performed by the farm veterinarian in collaboration with the Clinical Ruminant Service (SCRUM), Faculty of Veterinary Medicine, University of Zaragoza. Image interpretation was carried out by a single evaluator who was blinded to clinical data, macroscopic findings, and histopathological results.

### 2.4. Slaughter, Necropsy Procedures, Histopathological Examination and Molecular Confirmation

All animals were slaughtered according to standard commercial procedures at a certified abattoir. Heads were collected immediately after slaughter and transported under refrigeration to the Faculty of Veterinary Medicine of Zaragoza. The interval between slaughter and necropsy did not exceed 6 h.

**Figure 2:**
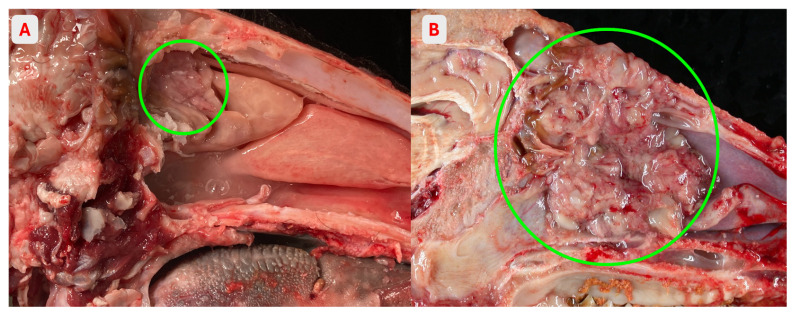
Sagittal section of the goat skull showing gross pathology of the enzootic nasal tumour in the nasal cavity. (**A**) Early-stage enzootic nasal tumor (within circle) in the ethmoidal turbinate. (**B**) Multinodular proliferative mass (within circle) occupying the nasal cavity and effacing the ethmoidal turbinates, coated by mucoid material.

Each head underwent systematic external examination followed by mid-sagittal sectioning to allow complete inspection of the nasal cavity (Figure 2). Particular attention was paid to the ethmoidal turbinates. Macroscopic lesions suggestive of neoplastic or inflammatory processes were recorded and photographically documented.

Representative tissue samples were collected bilaterally from the ethmoidal region in all animals, irrespective of the presence or absence of gross lesions. One tissue sample was obtained from each side to ensure standardised and comparable sampling across animals. Samples were fixed in 10% neutral-buffered formalin for at least 48 h and processed using routine paraffin-embedding procedures. Sections (4 µm thick) were stained with Carazzi’s haematoxylin and eosin.

**Figure 3:**
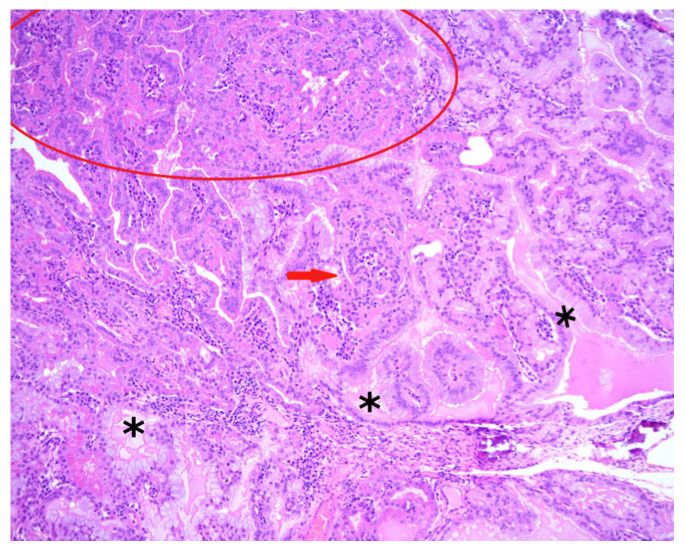
Histopathology of enzootic nasal adenocarcinoma. Neoplastic epithelial cells are arranged in mainly acinar (red circle) and tubular patterns, with occasional papillary projections (red arrow) into glandular lumina. The lumina contain eosinophilic secretory material (asterisks). Hematoxylin and eosin, 10× objective.

Histopathological evaluation was performed by the Histology and Pathological Anatomy Unit, Department of Animal Pathology, University of Zaragoza. The pathologist was blinded to thermographic findings and clinical data. Histopathological diagnosis was considered the reference standard for confirmation or exclusion of ENA (Figure 3). When gross lesions were present, multiple sections from the same tissue block were examined to minimise the risk of false-negative results.

For confirmation of ENTV-2 infection, histologically positive samples were frozen and subsequently submitted for specific detection of viral RNA by RT-PCR using the commercial kit EXOone Caprine Enzootic Nasal Tumour (EXOPOL SL, Zaragoza, Spain).

### 2.5. Statistical Analysis

Descriptive statistics were used to characterise the study population. Diagnostic performance of infrared thermography and macroscopic examination was assessed by calculating sensitivity, specificity, positive predictive value (PPV), negative predictive value (NPV), and overall diagnostic accuracy, using histopathology as the reference standard. A 2 × 2 contingency table was constructed to derive the number of true positives (TP), false positives (FP), false negatives (FN), and true negatives (TN). Sensitivity was defined as TP/(TP + FN), specificity as TN/(TN + FP), PPV as TP/(TP + FP), NPV as TN/(TN + FN), and overall diagnostic accuracy as (TP + TN)/(TP + FP + FN + TN). For each proportion, 95% confidence intervals were estimated using binomial methods appropriate for diagnostic test evaluation.

Agreement between diagnostic techniques was evaluated using Cohen’s Kappa coefficient and interpreted according to the criteria proposed by Landis and Koch. Statistical significance for agreement analysis was set at *p* < 0.05. All analyses were performed using IBM SPSS Statistics version 28.0 (IBM Corp., Armonk, NY, USA).

## 3. Results

### 3.1. Thermographic, Macroscopic, Histopathological and Molecular Findings

Thermographic assessment identified a total of 25 animals as compatible with ENA (29.1%), whereas 61 goats showed no thermographic evidence suggestive of the condition (70.9%).

Following slaughter and sagittal sectioning of the head for macroscopic examination, 21 animals (21.4%) exhibited an ethmoidal turbinate mass compatible with ENA, while the remaining 65 goats showed no macroscopic evidence of tumour growth (78.6%).

Histopathological examination of the ethmoidal region was successfully performed in all 86 goats and served as the reference standard for ENA diagnosis. Animals were classified as ENA-positive or ENA-negative based on the presence or absence of adenocarcinoma arising from the nasal glandular epithelium.

A total of 23 goats (26.7%) were diagnosed as ENA-positive, whereas 63 animals (73.3%) showed no histological evidence of ENA. All histologically positive cases were subsequently confirmed by RT-PCR for ENTV-2 (Table 1).

**Table 1:** Comparative summary of thermographic, macroscopic, and histopathological findings in the goats of the study (*n* = 86).

Diagnostic Method	ENA-Positive (*n*, %)	ENA-Negative (*n*, %)
Infrared thermography	25 (29.1%)	61 (70.9%)
Macroscopic examination	21 (21.4%)	65 (78.6%)
Histopathology (reference standard)	23 (26.7%)	63 (73.3%)

### 3.2. Diagnostic Performance Using Histopathology as the Gold Standard

The diagnostic performance of infrared thermography and macroscopic examination was evaluated using histopathological diagnosis as the reference standard. Sensitivity, specificity, positive predictive value (PPV), negative predictive value (NPV), and overall accuracy are summarised in Table 2.

Infrared thermography showed a sensitivity of 82.6% and a specificity of 90.5%, with an overall diagnostic accuracy of 88.4%. The PPV and NPV were 76.0% and 93.4%, respectively.

Macroscopic examination demonstrated a sensitivity of 82.6% and a specificity of 96.8%, with an overall accuracy of 93%. The PPV and NPV were 90.5% and 93.8%, respectively.

### 3.3. Agreement Between Diagnostic Techniques (Kappa Coefficient)

Agreement between thermographic evaluation, macroscopic examination, and histopathological diagnosis was assessed using Cohen’s Kappa coefficient. Pairwise comparisons are summarised in Table 3.

The highest level of agreement was observed between macroscopic examination and histopathology (κ = 0.817), corresponding to almost perfect agreement. Agreement between thermographic evaluation and histopathology was also substantial (κ = 0.711). Similarly, agreement between thermography and macroscopic examination reached a substantial level (κ = 0.704).

## 4. Discussion

The present study provides clinically relevant evidence supporting the use of infrared nasal thermography as an antemortem screening tool for ENA in goats under field conditions. Given the recognised difficulties in early ENA diagnosis and the absence of reliable serological tests [1,12], the identification of rapid and non-invasive diagnostic alternatives is of practical importance for herd-level disease management.

As expected, macroscopic examination showed the highest agreement with histopathology (κ = 0.817), confirming that once ENA lesions become macroscopically evident, gross pathological assessment is highly reliable. ENA originates in the secretory epithelium of the ethmoidal glands and progressively replaces turbinate structures, leading to obstruction and architectural distortion of the nasal cavity [9,18,19]. However, both macroscopic and histopathological evaluations are post-mortem procedures and therefore unsuitable for real-time herd control strategies.

Infrared thermography demonstrated substantial agreement with histopathology (κ = 0.711), high specificity (90.5%), and good overall accuracy (88.4%). Direct comparison between thermography and macroscopic examination should be interpreted with caution, as both methods are applied under fundamentally different conditions (antemortem vs. post-mortem). Thermography is inherently non-invasive and applicable under antemortem conditions, and its role should therefore be considered complementary rather than substitutive, particularly as a preliminary screening approach.

The relatively small number of ENA-positive animals (*n* = 23) resulted in wide confidence intervals, indicating uncertainty in the estimated diagnostic performance, particularly for sensitivity and positive predictive value.

The observed unilateral focal hyperthermia and thermal asymmetry in ENA-positive goats are pathophysiologically plausible. Neoplastic transformation is associated with increased metabolic activity, angiogenesis, and vascular remodelling, contributing to local heat production [20,21]. In addition, tumour-associated inflammation and obstruction of airflow may further modify surface temperature distribution. In healthy animals, symmetrical airflow contributes to balanced evaporative cooling. Partial or unilateral obstruction in ENA-affected goats may disrupt this equilibrium, enhancing detectable thermal asymmetry. Comparable mechanisms have been described in obstructive and inflammatory respiratory conditions in small ruminants [15,17].

Infrared thermography has been applied in veterinary medicine to detect inflammatory and respiratory disorders in ruminants [22,23,24,25]. The present study extends these applications by focusing specifically on the ethmoidal region, the primary anatomical site of ENA development, thereby increasing disease specificity compared with broader cranial thermal assessments.

The high specificity observed is particularly relevant for herd management, as it reduces the likelihood of false-positive classifications and unnecessary culling. The strong negative predictive value further supports its utility for excluding unaffected animals in herds with variable ENA prevalence [1,8]. Nevertheless, the moderate sensitivity indicates that small, deeply located, or minimally vascularised lesions may not generate sufficient surface thermal changes for detection. This limitation is consistent with thermographic literature, where lesion depth and vascularity influence detectability [20,21].

Thermographic findings in animals must be interpreted within the framework of differential diagnoses, as infrared thermography detects superficial temperature variations without etiological specificity [26]. Inflammatory processes in goats have been shown to induce localized hyperthermia detectable by thermography, as demonstrated in mastitis cases [27]. Therefore, inflammatory or traumatic nasal conditions may similarly produce localized thermal increases, potentially leading to false-positive interpretations when assessing nasal neoplasia. Integration with thorough clinical examination and, where possible, longitudinal monitoring may improve diagnostic confidence and specificity.

Several limitations should be acknowledged. The present findings should be interpreted as preliminary evidence of diagnostic accuracy, as the study relied on a single-herd, cull-population design. This approach may not fully reflect the variability encountered under routine field screening conditions. Furthermore, the use of a culled population may have overrepresented advanced cases, potentially influencing diagnostic performance estimates and limiting the generalizability of the results.

Regarding the methodology, thermographic interpretation was performed by a single blinded evaluator using qualitative criteria. Consequently, intra- and inter-observer agreement were not assessed, and the reproducibility of the proposed classification remains unknown. This restriction affects the immediate clinical applicability of the method and highlights the critical need for standardized interpretation criteria. Future multicentre studies, incorporating different breeds, production systems, and environmental conditions, are required to establish robust protocols and confirm the reproducibility of thermography as a diagnostic tool.

## 5. Conclusions

In conclusion, infrared nasal thermography represents a promising preliminary approach for antemortem assessment of ENA in goats. Although it cannot replace histopathological confirmation, it provides a practical antemortem approach that may facilitate earlier identification of suspect animals and support herd-level control strategies.

## Figures and Tables

**Table 2:** Diagnostic performance of each technique compared to histopathology as the gold standard (95% CIs).

Technique	Sensitivity (%)	Specificity (%)	Accuracy (%)	PPV (%)	NPV (%)
Thermography	82.6 (61.2–95.0)	90.5 (80.4–96.4)	88.4 (79.7–94.3)	76.0 (54.9–90.6)	93.4 (83.8–98.2)
Macroscopic examination	82.6 (61.2–95.0)	96.8 (89.0–99.6)	93 (85.4–97.4)	90.5 (69.6–98.8)	93.8 (85.0–98.3)

**Table 3:** Pairwise Cohen’s Kappa coefficients between diagnostic techniques for ENA in goats. Interpretation: 0.41–0.60 = moderate agreement; 0.61–0.80 = substantial agreement; >0.81 = almost perfect agreement. All Kappa values were statistically significant (*p* < 0.001).

Compared Techniques	Thermography	Macroscopic Examination	Histopathology
Thermography	–	0.704	0.711
Macroscopic Examination		–	0.817
Histopathology			–

## Data Availability

The original contributions presented in this study are included in the article. Further inquiries can be directed to the corresponding author.
